# Complex Electromagnetic Issues Associated with the Use of Electric Vehicles in Urban Transportation

**DOI:** 10.3390/s22051719

**Published:** 2022-02-22

**Authors:** Krzysztof Gryz, Jolanta Karpowicz, Patryk Zradziński

**Affiliations:** Laboratory of Electromagnetic Hazards, Central Institute for Labour Protection—National Research Institute (CIOP-PIB), Czerniakowska 16, 00-701 Warszawa, Poland; krgry@ciop.pl (K.G.); jokar@ciop.pl (J.K.)

**Keywords:** electromagnetic field, exposure, electric vehicle, urban transportation, environmental engineering, electromagnetic exposure, electromagnetic compatibility

## Abstract

The electromagnetic field (EMF) in electric vehicles (EVs) affects not only drivers, but also passengers (using EVs daily) and electronic devices inside. This article summarizes the measurement methods applicable in studies of complex EMF in EVs focused on the evaluation of characteristics of such exposure to EVs users and drivers, together with the results of investigations into the static magnetic field (SMF), the extremely low-frequency magnetic field (ELF) and radiofrequency (RF) EMF related to the use of the EVs in urban transportation. The investigated EMF components comply separately with limits provided by international labor law and guidelines regarding the evaluation of human short-term exposure; however other issues need attention—electromagnetic immunity of electronic devices and long-term human exposure. The strongest EMF was found in the vicinity of direct current (DC) charging installations—SMF up to 0.2 mT and ELF magnetic field up to 100 µT—and inside the EVs—up to 30 µT close to its internal electrical equipment. Exposure to RF EMF inside the EVs (up to a few V/m) was found and recognized to be emitted from outdoor radiocommunications systems, together with emissions from sources used inside vehicles, such as passenger mobile communication handsets and antennas of Wi-Fi routers.

## 1. Introduction

### 1.1. Electric Vehicles and Their Technical Infrastructure

For ecological and economic reasons, electric traction vehicles (ETV), (trams, metro, trolleys and railway commuter trains) are an important methods of public transportation in urban areas and for intercity connections. These ETVs are powered by stationary sources of electricity (direct current (DC) or alternating current (AC) electric traction installations) that are directly connected to vehicles through wires. Technological developments also make it possible to use electric energy far from electric traction lines in motor vehicles as in passenger cars (e.g., taxis as a form of public transport, but also private vehicles) and buses (e-mobility technology) in various configurations of driving and supplying systems:

- a hybrid electric vehicle (HEV)—type of hybrid vehicle that combines a conventional internal combustion engine with electric propulsion to achieve either better fuel economy than a conventional vehicle or better performance;
- a plug-in hybrid electric vehicle (PHEV)—a type of hybrid vehicle whose battery can be recharged by plugging it into an external source of electric power, as well as by an on-board conventional internal combustion engine and generator;
- a plug-in electric vehicle (PEV)—any type of motor vehicle that can be recharged from an external source of electricity, such as wall sockets, where the electricity stored in the rechargeable battery drives the wheels;
- a fuel-cell electric vehicle (FCEV)—the electric engine is powered by electricity generated in the fuel cells by a chemical reaction of compressed hydrogen (from the tank in the vehicle) and oxygen from the air.

Forecasts indicate further development of these types of motor vehicles, and an increase in their contribution to public transport. For example, in Europe in 2019, around 2300 electric buses were used. Forecasts for 2025 indicate a ten-fold increase [1].

The operation of PEV or PHEV is inseparably connected to the need to charge electricity sources in the vehicles (using batteries or highly efficient capacitors known as supercapacitors) by wire (in charging stations) or wirelessly (using systems located under the road surface). In 2020 in Europe, approximately 200,000 public charging stations were used, of which 24,900 were fast charging stations with power exceeding 22 kW [2].

Charging may be performed by developed systems of wireless power transfer (WPT), in which power is transferred via EMF of frequency approx. 20–100 kHz, or in the traditional way by charging a battery with DC current using wired installations.

**Figure 1:**
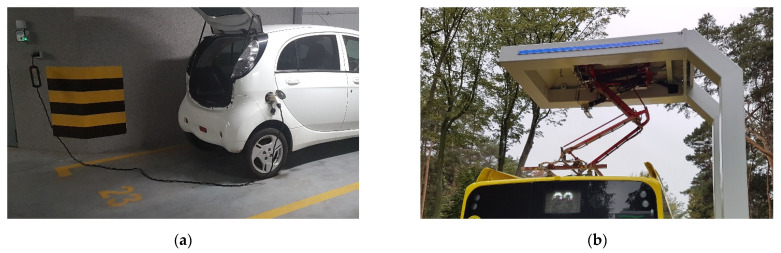
Charging of PEVs with the use of: (**a**) slow AC charger and cable connected to the socket in the passenger car (AC/DC rectifier inside car); (**b**) fast DC charger station (AC/DC rectifier outside car) and pantograph system (source: the authors’ collection).

There are two traditional forms of charging process and charging station: slow and fast. In slow charging stations, with a power supply typically from 1.5 kW to 22 kW (i.e., with current ranging 6–90 A), the batteries in PEVs are charged via a cable connected to a socket with AC of 50 Hz from a single-phase 230 V source (including typical electrical installation used by electricity consumers, i.e., at home) or a three-phase 400 V installation with a maximum phase current of 32 A (Figure 1a). In this case, PEVs use AC/DC converter systems (rectifiers) located inside vehicles.

In fast charging stations, with power exceeding 22 kW and high-power charging with power exceeding 100 kW (up to 350 kW, which allows several vehicles to be charged simultaneously, reducing the charging time by several hundred minutes, depending on the capacity of the batteries and their state of charge), the batteries in PEV are charged using an AC of 50 Hz converted to a DC (rectified) current of up to 400 A and a voltage of up to 1000 V, via a cable connected to the socket, or pantographs attached to the rails located on a bus roof, e.g., in a depot or a final stop (Figure 1b).

### 1.2. Electromagnetic Field Inside EV

#### 1.2.1. Static Magnetic and Extremely Low-Frequency Electromagnetic Field

The use of these electric vehicles (EVs) in public transportation, including ETVs, HEVs, PHEVs and PEVs, results in the emission of electromagnetic fields (EMF) with a complex frequency spectrum due to the diverse designs of electric drive systems, the variability of power supply parameters while driving, and the use of various electrical equipment, e.g., low-voltage 600 V or 750 V DC current bus bars or cables located along the paths of ETVs (overhead in trams, trolleys and commuter trains, but under the floor in metros) and supplying DC or AC driving engines. Those outdoor DC power lines emit static and extremely low-frequency (ELF) EMF at fundamental frequencies usually of 50 and 300 Hz, or 300 and 600 Hz together, with a set of higher-frequency harmonics (coming from the imperfect rectification of 50 Hz AC currents from single-phase or three-phase installations). Despite the DC outdoor traction system, ETVs and PEVs are very often equipped with AC engines. Consequently, they need to use DC/AC power inverters located in various places (e.g., on the roof, under the floor or in passenger sections), sometimes located at some distance from the engine and connected by cables.

Driving systems (typically AC engines) and their supplying internal installations are the main sources of ELF EMF. The engines used in EVs are usually located under the floor (metro, trams), or in the rear of the passenger section (trolleys, buses). Exposure to the electric component of ELF EMF (electric field—EF) may be treated as negligible because of the use of low voltage and the metal construction of the vehicles and housing of the indoor devices, electromagnetically shielding EV inner space and reducing this component of EMF inside EV.

**Figure 2:**
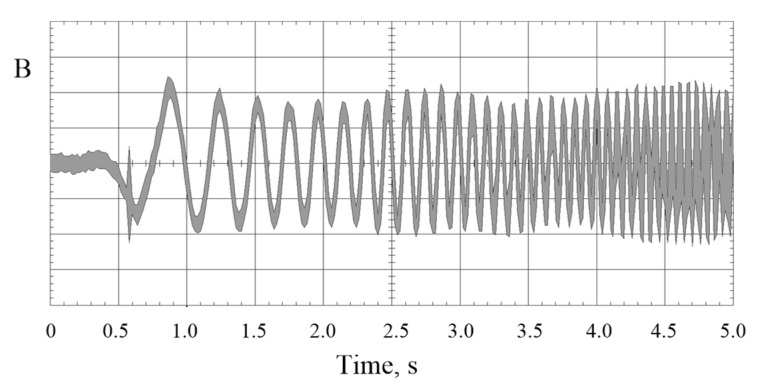
The magnetic field waveform recorded in the vicinity of installations supplying driving engines inside a plug-in electric vehicle (PEV) type of bus; illustrative non-calibrated recordings using magnetic field flux density (B) probe of 1–400 kHz flat frequency response (source: the authors’ collection).

The magnetic component (magnetic field—MF) of EMF is not shielded by the vehicle construction and penetrates the driver’s cabin and passenger areas. Time- and frequency-domain characteristics of MF emitted by electric power supply installations and engines depend on changes to the mode of driving and changes to the installation power load. Figure 2 shows waveforms characterizing MF changes.

**Figure 3:**
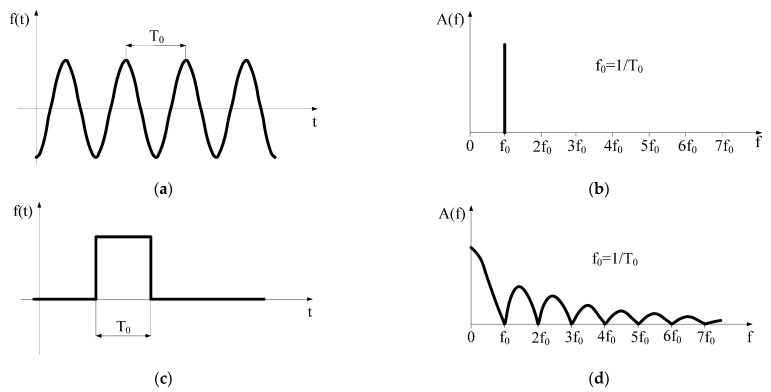
The frequency spectrum of sinus waveform (**a**,**b**) and single rectangular pulse (**c**,**d**) (illustration on the base of Fourier transform principles).

Our investigations showed that the frequency spectrum of ELF EMF emitted by engines and supply equipment in EVs covers a range from several Hz up to 300 Hz, with dominant components usually in the several tens of Hz [3]. Amplitudes of components at frequencies exceeding 300 Hz are usually lower than 5% of the fundamental frequency. According to published data, the maximum levels of recorded magnetic field were found to be 50 Hz in a tram, 15.3–16.5 Hz in a train, and 12 Hz in a hybrid car [4]. It must be remembered that compared to power frequency ELF EMF (characterized by a single harmonic component of 50/60 Hz) emitted by the regular AC electric power installations in houses or offices, or by overhead high-voltage transmission lines, in the EVs the EMF spectrum frequently includes a static MF component (SMF) from the DC component of currents, and its time-varying component is characterized by a fundamental frequency that may be different to 50/60 Hz (both lower or higher), and may include higher harmonics in MF (which, in the frequency domain, may be represented as a wide-band spectrum of harmonics—Figure 3).

The charging systems of PHEVs or PEVs emits complex EMF, specifically static and ELF with dominant frequencies at 50 and 300 Hz as a result of pulsation in a rectified three-phase current. The level of the charging DC current and SMF varies during the charging process, and depends on the state of the battery charge—it is highest when beginning the charging, and then decreases over time.

#### 1.2.2. Radiofrequency Electromagnetic Field

Modern vehicles (and EVs in particular) are equipped with more and more modern wireless communication technologies working as Internet of Things (IoT) systems, e.g., passive keyless entry (PKE), device-to-device (D2D), intra-vehicle (InV), vehicle-to-vehicle (V2V), vehicle-to-infrastructure (V2I), vehicle-to-everything (V2X) communications or vehicular ad-hoc network (VANET) [5,6]. For this purpose, several technologies, protocols and standards for wireless communication are used, such as: wireless fidelity (Wi-Fi), mobile communication, long-term evolution (LTE), Bluetooth, worldwide interoperability for microwave access (WiMAX), wireless access in vehicular environments (WAVE), dedicated short-range communications (DSRC), radio frequency identification (RFID), near-field communication (NFC), ZigBee, etc.

The cabins of EVs also have radio communication equipment, e.g., short-distance radio communication facilities used for local systems of wireless access to the Internet using Wi-Fi technology (recognized as routers equipped with transmitting–receiving antennas), which are further sources of radiofrequency (RF) EMF operating in the industrial, scientific, and medical (ISM) radio frequency bands (Wi-Fi 2G: 2.40–2.48 GHz and Wi-Fi 5G: 5.15–5.73 GHz) [7]. In addition, there are handsets of public mobile communication systems used by passengers, which are sources of RF EMF from the uplink (UL) frequency bands used in communication of handsets to base transceiver stations (BTS) (GSM: 880–915 MHz, DCS: 1710–1785 MHz and UMTS: 1920–1980 MHz, LTE frequency bands differing across the world in the range 700–2700 MHz, e.g., in Europe LTE 800: 832–862 MHz (FDD—Frequency Division Duplex), LTE 1800: 1710–1785 MHz (FDD), LTE 2100: 1920–1980 MHz (FDD) and LTE 2600: 2500–2570 MHz (FDD) and 2570–2620 MHz (TDD—Time Division Duplex) [8].

**Figure 4:**
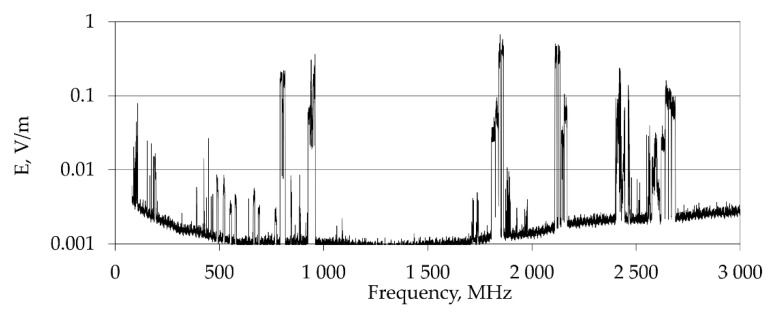
Examples of RF EMF frequency spectrum in the range (80–3000) MHz recorded in the center of a big city (source: the authors’ collection).

Exposure to RF EMF inside ground EV of public transportation (buses, trams, trolleys and trains) is correlated with the EMF component emitted by various outdoor communication systems (located outside vehicles) such as radio and television (RTV) broadcasting antennas (FM radio: 88–108 MHz and analogue and digital TV: 174–862 MHz), public mobile network BTS antennas providing the transmission of signals to handsets (DL—downlink band), using various mobile services—LTE 800: 791–821 MHz, GSM: 925–960 MHz, DCS: 1805–1880 MHz, UMTS and LTE 2100: 2110–2170 MHz, LTE 2600: 2620–2690 MHz) [9,10]. Figure 4 shows an example of the RF EMF spectrum recorded in the downtown of a city (Warszawa, Poland) including the components of exposure mentioned above.

### 1.3. Aim of Study

The issue of human and electronic device exposure to EMF associated with the use of EVs comes under new environmental EMF factors that need attention with respect to various professional groups (e.g., drivers), but also the general public (e.g., passengers using EVs daily). The application of wireless communication technologies and engines and supply equipment in EVs is related to the use of multiple EMF sources in a relatively small, limited space. This requires consideration of the following issues: (1) potential environmental health hazards to humans from short-term (exposure for durations less than the corresponding averaging time) and chronic, long-term (during a major part of the lifetime) EMF exposure (as considered by international authorities) [11,12,13]; and (2) interference malfunctions caused by an EMF disturbance that affects the performance of an electronic device recognized as electromagnetic interference (EMI) and characterized in relation to electromagnetic compatibility (EMC) [14,15,16]. An important element of this process is identifying the characteristics of EMF associated with the use of EVs, assessing its significance for human safety and health, as well as recognizing the need to implement relevant preventive measures aimed at decreasing various electromagnetic hazards [17].

The aim of this study was to recognize and evaluate exposure to EMF associated with the use of EVs in urban transportation.

## 2. Materials and Methods

### 2.1. SMF Measurements

Spot measurements of SMF in urban transportation EVs (near the indoor equipment supplied by DC) and in the vicinity of charging stations, at a minimum distance of 2 cm away from housing and cables, were performed with the use of the SMF-sensitive 3-axis (isotropic) Hall-probe magnetic flux density (B) meter MF THM1176-MF (Metrolab Instruments SA, Geneva, Switzerland), with measurement range of 0.1–3000 mT, resolution 0.1 mT, and accuracy ±1%.

### 2.2. ELF EMF Measurements

Measurements of MF inside various types of EVs of urban transportation (ETV: trams, trolleys and PEV, HEV: buses, passenger cars) and in the vicinity of charging stations were taken with the use of devices sensitive to root-mean-square (RMS) value of B (B_RMS_):

- EFA-300 (Narda Safety Test Solutions, Pfullingen, Germany)—with 100 cm^2^ isotropic probe (according to IEC 61786-1 standard requirements [18]), with a frequency response range of 5–32 kHz (flat), measurement range 0.1 µT–32 mT, resolution 0.01 µT and accuracy ±3%.
- exposimeter EMDEX II Standard (Enertech Consultans, Campbell, CA, USA)—with isotropic probes and a frequency response range of 40–800 Hz, measurement range 0.01–300 µT, resolution 0.01 µT, accuracy ±2% and sampling rate 1.5 s.

The spot measurements in the vicinity of charging stations or electrical equipment inside the EV covered spatial distribution of MF from cables and housing, at a minimum distance of 10 cm.

The data logger investigations of exposure in trams, trolleys, and buses were carried out near the recognized ELF MF sources (AC engine cables connecting DC/AC inverters with engines, etc.) located in various places inside the vehicles (e.g., at the rear of the vehicle in the section for passengers, or behind the driver’s cabin). The use of exposimeters is justified in the case of variation of MF level as in EVs, and explicitly recommended by IEC 61786-2 standard [19]. Data loggers were placed motionless on seats at a height of approx. 60 cm above the floor, close to the housing of electrical equipment (at a minimum distance of approx. 10 cm) or at a longer distance (several meters from the equipment). In passenger cars, data loggers were placed on the front and rear seats. ELF MF is not disturbed by the presence of people in the measuring area.

ELF MF in EVs was recorded during journeys including various drive modes (starting, accelerating, driving at a constant speed, braking), taking approx. 15–40 min.

### 2.3. RF EMF Measurements

Our investigations were performed using frequency-selective exposimeters EME SPY 121 (Satimo, Brest, France)—a portable, pocket-sized data logger sensitive to EF strength (E) in the frequency range from 88–2500 MHz, split into 12 predefined frequency measurement ranges, corresponding to the most common RF EMF applications in wireless communication systems. The sensitivity of the exposimeters in each individual frequency band is 0.05 V/m, and the measurement range is 0.05–10 V/m. The measurements were performed with a programmable sampling rate of 4 s. RF EMF (similar to ELF MF) was recorded during regular ground routine journeys inside PEV buses with data loggers located on seats in the middle part of the passenger section and in the driver’s cabin, in the case of buses equipped in internal Wi-Fi 2G routers located inside cabin. The distance of passengers from the exposimeter was in a range approx. 0.5–5 m. The number of people traveling by bus using wireless communication devices changed during regular journeys—it can be assumed that it was at least a dozen people (measurements were taken during peak hours).

### 2.4. EMF Evaluation Principless

Our investigations were focused on EMF exposure, to analyze whether it may be significant with respect to human exposure evaluated with respect to international safety guidelines.

According to the International Commission on Non-Ionizing Radiation Protection (ICNIRP) guidelines, the limit value for the exposure of the general public to SMF is 400 mT [20]. International labor law, as well as ICNIRP guidelines, also provide for higher limits for worker exposure, but they are applicable on a temporary basis during the shift, when justified by the practice or process, provided that preventive measures, such as controlling movements and providing information to workers, have been adopted, and that periodic inspections of the exposure parameters are performed on a regular basis [17,20]. Taking into account the broad range of provisions attached to the use of limits on occupational EMF exposure, in the case of evaluating EMF exposure in EVs, they seem not to be relevant, and limits set for public exposure are discussed here in the context of reported results of environmental measurements. It should also be pointed out that potential health and safety hazards caused by indirect exposure effects observed in SMF may affect workers and the public at the same level of exposure, such as interference with active implanted devices (e.g., cardiac pacemakers) observed in SMF exceeding 0.5 mT, and a projectile risk from ferromagnetic objects observed in SMF exceeding 3 mT [17,20].

According to ICNIRP 2010 safety guidelines for the general public (exposure limits aimed at protecting against the electrostimulation of human tissues caused by the EMF-induced electric fields within them) the limits of exposure to ELF MF set with respect to its root-mean-square level (B_RMS_) decreases along with frequency (i.e., limits decrease from 625 µT up to 200 µT along the frequency range of 8–25 Hz, then are set at a fixed level of 200 µT in the frequency range 25–400 Hz, and again decrease from 200 µT to 27 µT along the frequency range 400–3000 Hz) [13]. In practice, when higher harmonics in the evaluated EMF are considered, the assessment of exposure is stricter, due to the abovementioned limits decreasing along with frequency.

According to the safety guidelines for general public exposure to RF EMF, exposure limits aimed at protecting against thermal effects in the exposed human body were set at 28 V/m in the frequency range of 10–400 MHz, increasing along the range 28–61 V/m in the frequency range 400–2000 MHz, to continue at a fixed level of 61 V/m in the range 2000–300,000 MHz, following Council Recommendation 519/1999/EC (1999) [21]. Following ICNIRP (2020), only a constant exposure limit for power density was provided for frequencies exceeding 2000 MHz [22].

Considerable attention must be focused on the longer daily exposure to workers, compared to passengers, and this is especially justified in the context of the results of studies on potential environmental health hazards from chronic EMF exposure [11,23].

Typical EMC immunity test levels are specified in non-obligatory international standards, applicable following manufacturer decisions or any specific market requirements: regarding radiofrequency EMF at 1, 3, 10 or 30 V/m as specified in, e.g., IEC 61000-4-3:2020, regarding power frequency (50 Hz, 60 Hz) EMF at 1, 3, 10, 30 or 100 A/m (1.25, 3.75, 12.5, 37.5 or 125 μT) as specified in IEC 61000-4-8:2009, regarding 9–150 kHz EMF at 1, 3, 10 or 30 A/m (1.25, 3.75, 12.5 or 37.5 μT) and regarding 150 kHz–26 MHz EMF at 0.1, 0.3, 1 or 3 A/m (0.125, 0.375, 1.25 or 3.75 μT) as specified in not-obligatory IEC 61000-4-39:2017 [14,15,16]. However, test levels specified in the particular product standard applicable for particular types of electronic devices may be different from these, and should be considered in adequate test procedures. Because of various practical reasons, many low-cost electronic components and devices are compliant with EMC requirements only at the low level the abovementioned EMF test exposure, e.g., their immunity to influencing 50 Hz EMF is sufficient up to a level of 1 A/m (1.25 μT), but this is not sufficient when the affecting EMF is several times higher or stronger.

## 3. Results

### 3.1. SMF Exposure in EVs and in the Vicinity of Charging Stations

Our investigation, performed while running or stationary while charging an EV, showed that SMF inside an EV is usually at a level below approximately 0.10 mT, with the maximum level not exceeding 0.2 mT near (at a distance of up to 2 cm away) indoor equipment supplied by DC (cables, battery pack). At the same distance in the vicinity of charging stations (power supplies cables) with maximum output current of up to approx. 300 A while charging, SMF does not exceed 0.2 mT.

### 3.2. ELF MF Exposure in the Vicinity of Charging Stations

Broadband (5 Hz–32 kHz)-measured RMS values of ELF MF component (with recognized dominant frequencies 50 and 300 Hz) do not exceed 20 µT at a distance of 10 cm away from the housing of stations and cables while charging with the maximum output current of up to 300 A (in the case of a fast DC charging station).

### 3.3. ELF MF Exposure in EVs

Table 1 summarizes the parameters of exposure to ELF MF obtained from exposimetric measurements of B_RMS_ inside various types of EV of urban transportation (ETV: trams, trolleys, PEV buses, PEV and HEV passenger cars) while routine running (covered starting, accelerating, driving at a constant speed, braking). Broadband (40–800 Hz)-recorded RMS values of ELF MF components do not exceed median values of the level of several microtesla (the highest measurement results were recorded in PEV buses up to 2.6 µT close to DC/AC equipment in PEV buses), whereas the recorded maximum values were ten times higher (the highest values were recorded in trolleys up to 33 µT). Recognized dominant frequencies of ELF MF in various EVs did not exceed 300 Hz.

### 3.4. RF EMF Exposure Inside EVs

**Figure 5:**
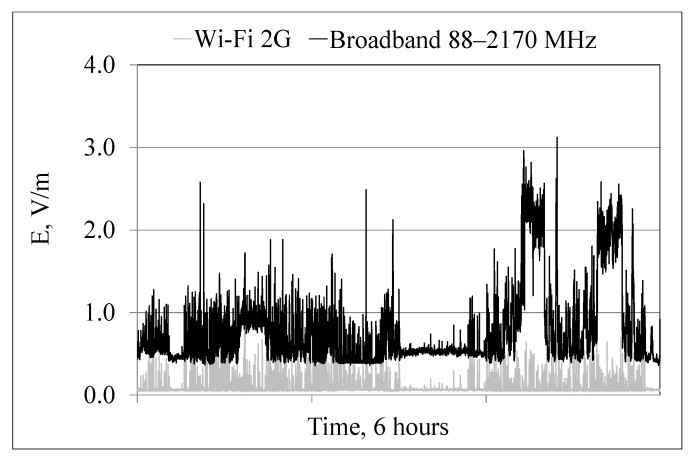
Variability of electric field strength (E) in the frequency band 88–2170 MHz, recorded in the driver’s cabin of a PEV bus with indoor Wi-Fi 2G router located on the ceiling while the journey with passengers over the city downtown (black line—total broadband exposure; grey line—narrow-band component from the internal Wi-Fi 2G equipment), (source: the authors’ collection).

**Figure 6:**
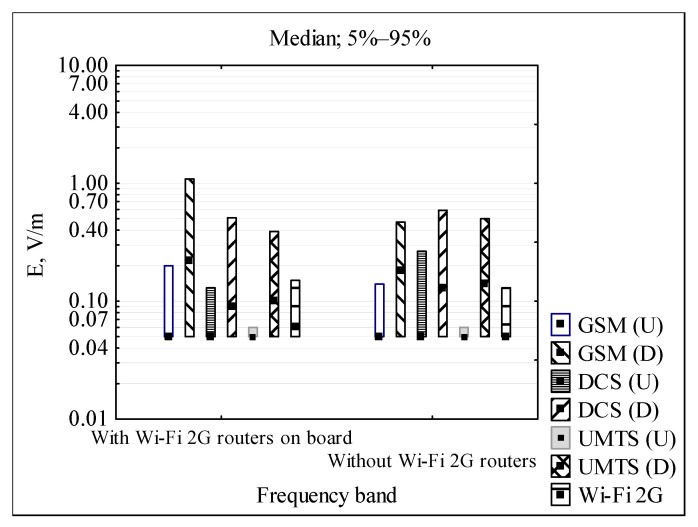
Statistical parameters of narrow-band frequency components of electric field strength (*E*) recorded in the passenger section of PEV buses equipped in indoor Wi-Fi 2G routers or without Wi-Fi routers during journeys with passengers through the city downtown; GSM (U) and GSM (D)—GSM 900 uplink and downlink, respectively; DCS (U) and DCS (D)—DCS 1800 uplink and downlink, respectively; UMTS (U) and UMTS (D)—UMTS 2100 uplink and downlink, respectively (source: the authors’ collection).

Figure 5 and Figure 6 illustrate exposure to RF EMF inside a PEV bus, with and without Wi-Fi 2G routers located on the ceiling of the driver’s cabin, during a routine drive through the downtown areas of the city.

Figure 5 shows the level of exposure to RF EMF emitted by an indoor Wi-Fi 2G antenna and other RF EMF outdoor and indoor sources (representing the broadband measurement results covering 11 frequency components, except Wi-Fi 2G) in the driver’s cabin. The obtained results show that the level of exposure is determined mainly by outdoor sources located in the city along the vehicle route (FM, TV, and especially BTS mobile communication GSM, DCS and UMTS downlink signals) and, to a lesser extent, by passenger mobile handsets.

Figure 6 presents the contribution of various types of RF EMF sources in the exposimetric profiles recorded in the passenger section of urban PEV buses, split into those equipped with indoor Wi-Fi 2G routers and those without routers. Taking into account the medians and the range between the 5th and the 95th percentiles of the recorded EF strength, in both cases the main components of exposure are BTS antennas of outdoor public mobile networks. The exposure from passenger mobile handsets (uplink components) and Wi-Fi 2G is lower. The variability of exposure to EF in individual frequency bands is the result of the irregular BTS locations along vehicle routes and actual traffic operated by them (regarding downlink signals), and changes to the number of passengers using mobile handsets and the different distance of passengers to the location of data loggers (regarding uplink signals). The level of exposure to RF EMF emitted by outdoor sources is similar to the exposure recorded inside buildings in an urban area [9].

## 4. Discussion

### 4.1. SMF Exposure in EVs and in the Vicinity of Charging Stations

Our investigation showed that SMF inside EVs is usually slightly higher than natural geomagnetic SMF, which is in the range of approx. 0.025–0.060 mT, depending on geographical location. Near industrial or medical devices, SMF affecting workers may even exceed 1000 mT near magnetic resonance imaging (MRI) scanners [24,25,26,27], 20 mT near aluminum production installations, and 5 mT near magnet production facilities and MIG/MAG welding cables [28].

The highest exposure to SMF found in connection to the use of EVs, up to 0.2 mT, may exist near traditional DC charging installations (charger unit and cables) and may be treated as negligible with respect to the abovementioned (Section 2.4) exposure limits, including stricter limits for persons with implanted electronic medical devices [17,20]. In short, the SMF component of EMF measured inside or near EVs is weak compared to SMF in other work environments, as well as compared to the limits of human exposure provided by international safety guidelines.

### 4.2. ELF EMF Exposure in the Vicinity of Charging Stations

DC charging installations are also sources of ELF MF emitted by a current supplying input circuit of 50 Hz AC and a current in an output DC circuit as a result of pulsation in a rectified three-phase current. The published results of investigations covering ELF MF (25 Hz–2 kHz) emitted by five DC fast charging stations with an output power of 20–120 kW showed that the maximum values of B_RMS_ measured in the worst exposure scenario, with high currents while charging between 10% and 50% of the state of battery charge, do not exceed 2–112 µT at a distance of 7.5 cm and 0.8–13 µT at a distance of 20 cm from the housing of the charging stations [29]. Spectrum analysis showed a dominant component of the EMF exposure was a frequency of 50 Hz. The level of B_RMS_ close to the charging stations is compliant with the safety limits of exposure (for example, according to ICNIRP 2010 safety guidelines for the general public, 200 µT at 50 Hz) [13]. Considering the higher harmonics in the output current (up to several hundred Hz emitted because of a technically imperfect current rectification), the assessment of exposure may be stricter, due to limits decreasing along with frequency. However, the measurement results remain compliant with the exposure limits.

### 4.3. ELF EMF Exposure in EVs

The results of other existing studies of the exposure to ELF MF in passenger cars (HEV, PHEV and PEV) have showed a higher level of MF in these vehicles, in comparison to combustion-powered cars. For example, Halgamuge et al. reported that the average maximum value of B_RMS_ of ELF MF measured in one HEV (data logger: frequency response 40–800 Hz and 3 s sampling rate) does not exceed 2.5 µT on the seats and 3.5 µT close to floor [4]. The maximum values of B_RMS_ were observed to be 12 Hz. In other studies, Hareuveny et al. reported ELF MF measurements (data logger: frequency response 40–800 Hz and 1.5 s sampling rate) in four diesel cars, four gasoline cars, and three HEVs under several conditions: idle mode and driving at variable speeds of 40 to 80 km/h [30]. The maximum values of B_RMS_ do not exceed 0.8 µT (median values 0.2 µT) in HEVs and 0.2 µT (0.15 µT) in other vehicles. Near the floor, B_RMS_ reached 10 μT.

Vassilev et al. investigated MF at frequencies of up to 10 MHz inside five PEVs, two HEVs, one FCEV, two gasoline passenger cars, and one diesel passenger car [31]. The measurements were taken while the cars were being driven, on the front passenger seat. Values of B_RMS_ ranging between 0.1 and 2 μT, at frequencies between a few Hz and 1 kHz, but less than 0.1 μT above 1 kHz, were measured. Close to the battery, the ratio between the magnetic flux density of SMF and the traction current was in the range 0.2–1 μT/A, depending on the car. Given that traction current has variations of up to 300 A, the SMF can reach approx. 300 μT close to the battery, which should be taken into consideration with respect to EMF exposure in the car services, but without much practical significance in terms of the passenger and driver exposure.

Pääkkönen et al. measured ELF MF (5–4000 Hz) in four PEVs, one HEV and two gasoline passenger cars during an urban drive, with the speed varying from 40 to 70 km/h [32]. The maximum values of B_RMS_ measured on seats do not exceed 2.6 µT in PEVs and HEVs, and 2.2. µT in gasoline cars. The highest values of B_RMS_ in PEVs were obtained in the 8–10 Hz frequency range.

Yang et al. investigated MF in three PEV passenger cars over a period of two years (2017–2019) to check the influence of changing with replacement of the components or maintenance on level of exposure [33]. The broadband measurements (1 Hz–2 kHz) were performed in the front and rear passenger seats during acceleration and while driving at a constant speed. The maximum mean B_RMS_ values do not exceed 1.6 µT. It was found that the variation of the major spectral components of MF was larger for repaired cars (the use of spare components or inappropriate mounting of components during repair), compared to the results from the cars with regular maintenance.

Summarizing the presented research results, it can be concluded that the maximum component of exposure to ELF MF inside EVs (at a frequency of up to 300 Hz) does not exceed the B_RMS_ value of 30 µT, close to electrical equipment (DC/AC inverters or cables connecting inverters with engines) and 3–4 µT at a longer distance. Significant differences were not found in the level of exposure depending on the type of vehicle (PEVs, HEVs, gasoline cars).

The evaluation of compliance of the recorded B_RMS_ values using data loggers of measurement frequency range of 40–800 Hz with exposure limits decreasing with frequency can be performed using a worst-case exposure scenario (assuming the highest dominant frequency of measured ELF MF) and the limits defined for such frequency (in the case of measurements in EVs—300 Hz). Reported measurement results in the abovementioned studies and reported in the abovementioned research literature are compliant with general public exposure limits.

In most of the abovementioned studies, commercially available broadband RMS-value magnetic flux density data loggers, with frequency response 40–800 Hz or similar measurement frequency band, were used. The abovementioned measurement devices allow the covering of dominant frequency components of steady-state exposure (components of magnetic field from a few Hz up to 300 Hz, recognized in the frequency spectrum of EMF emitted by engines and supplying installations, with dominant components in the several tens of Hz). However, it should be mentioned that these data loggers, being RMS-value measurement devices, are dedicated for investigating harmonic steady-state component of a magnetic field. Consequently, they have only limited use in the evaluation of parameters of transient or pulsed waveform magnetic fields. In previous studies, measurements of transient MF inside metro cars were systematically performed using a specially designed data logger with programmable frequency of sampling (1, 10, 100, or 1000 per second) equipped with isotropic MF probes [34]. In this study, it was found that using a sampling of at least 100 Hz, the obtained results of measurements in metro cars sufficiently characterize a range of MF variability. Using a slower sampling rate (e.g., 1 Hz, similarly to the sampling rate available when RMS-value data loggers are used), the transient component of MF is missing from the measurement results, and the recorded maximum value of MF can be underestimated by 2–3 times, compared to faster sampling (e.g., 100 Hz). Similar underestimation of maximum value of MF recorded by RMS-value data loggers is expected in the discussed results of measurements in EVs. Taking into account the abovementioned empirical evidence and reported results of RMS-value broadband recordings, it seems to be justified the opinion that the maximum value of magnetic flux density does not exceed 100 µT during transient short fluctuations caused by changes in mode of drive.

For assessment of MF exposure in transients, the guidelines provided by ICNIRP recommend using the weighted peak method [13], discussed for example during the assessment of the human exposure to MF generated by dynamic inductive power transfer systems for automotive applications in [35]. The abovementioned level of the highest recorded maximum values of ELF MF is compliant with ICNIRP guidelines regarding general public exposure [13].

### 4.4. RF EMF Exposure in EVs

Similar results to ours have been obtained in other investigations, for example one performed in Spain on buses of a public transport system, which used the similar type of frequency-selective exposimeter [36]. During the journey, several mobile voice connections were voluntarily performed, emulating passengers on the bus making a call, via three transmitters (GSM 900, DSC (GSM) 1800, UMTS 2100) located in the front, central and rear parts of the bus. Exposimeters were in the closest sitting place, within an area of 2 m around the user making the phone call. The measured EF exposure did not exceed 3 V/m. The highest levels of EF were produced by mobile communication systems (GSM/UMTS-U), initiated by mobile voice connections during measurements.

Frequency-selective exposimeter investigations performed in trams are also reported in [37]. The measurements covered various passenger densities (high density during peak hours in a normal business day, and low density with just a few people inside the tram). Similar to buses, the highest exposure—up to 4 V/m—was recorded there while using mobile communication systems (GSM/UMTS—U) and Wi-Fi 2G.

According to the safety guidelines for general public exposure to RF EMF, the exposure levels found in EVs are significantly lower than the limits of EF strength (as mentioned in Section 2.4).

The frequency-selective investigations of RF EMF show that, aside from the place of measurement, the maximum level of exposure inside ground public transportation EVs is caused mainly by the use of mobile handsets inside vehicles (EF strength up to 0.5 V/m as the median value, and up to 2 V/m in 95th percentile in the set of recorded values). Inside ground EVs, the profiles of exposure to RF EMF emitted by outdoor sources are similar to the profiles of exposure recorded in buildings in an urban area (dominating components of RF EMF emitted by RTV and BTS antennas and Wi-Fi routers).

### 4.5. Health Aspects of Exposure to EMF in EVs

An EV driver’s long-lasting daily exposure to EMF, even if compliant with the exposure limits, cannot be counted to be negligible when the context of possible adverse health effects due to chronic exposure to EMF is considered. The ELF MF was classified to be a possible carcinogenic to human (2B classification) based on the epidemiologically proven elevated carcinogenic health risks in populations chronically exposed to MF exceeding 0.4 μT (attention level related to yearly averaged exposure) [38,39,40]. The level of ELF MF exposure reported in various studies focused on EMF in EVs and discussed in this article may significantly contribute to the total long-lasting exposure to drivers.

The effects of EMF exposure induced in exposed objects are frequency-dependent, but the significant majority of studies performed so far in the area of EMF safety have referred to the populations exposed to high-voltage power lines (i.e., to chronic exposure to EMF of sinusoidal power frequency), and the outcome of such observations was a base for the abovementioned 2B classification for ELF MF exceeding 0.4 μT. Because of differences in the frequency patterns of the discussed exposures (near power lines and in EVs), there needs to be very careful analysis of how far the studied health and safety outcomes from ELF EMF exposures vary in such cases, and which exposure metrics are relevant to evaluate them. Consistently, the mentioned differences in frequency characteristics of ELF EMF in EVs and EMF near regular electric power installations also need attention with respect to the exposure evaluation protocol, which in practice means that studies of the parameters of EMF exposure associated with the use of EVs require not only measurements of the RMS value (which, in practice, is usually almost equal to the RMS value of the dominant frequency component of exposure), but also attention to the higher harmonics of this exposure, the components of fundamental frequencies other than 50 Hz, the parameters of transient EMF over rapid changes in the mode of EV driving, and combined exposure including the abovementioned components.

Similar to ELF MF, RF EMF was classified by the IARC in the group of 2B carcinogenic environmental factors [41]. This component of driver EMF exposure also needs attention because of its level at least comparable to office exposure, where wireless radio communication facilities are in use and daily long-lasting exposure, potentially significantly contributing to total driver chronic exposure, combines with other components of lower frequencies (covering together exposure to: static, low frequency and radiofrequency fields).

### 4.6. EMC Issues

The obtained results showed that the level of recorded ELF and RF EMF values in EVs exceed the lowest EMC immunity test levels of exposure (as mentioned in Section 2.4) especially near EMF sources. This must be taken into account, given the environmental impact to which devices used in EVs should be immune—only devices for which immunity has been confirmed at levels higher than the EMF found inside EVs (by applying EMF levels higher than the basic ones during EMC tests) should be used in EVs. This is very important for the proper operation of these devices and, consequently, for the safety of drivers and passengers.

## 5. Conclusions

In every urban area, there is a daily mass of passengers traveling by public transportation. Ecological and economic reasons, as well as technological development, mean that a significant percentage of the population already use EVs (trams, metro, trolleys, buses) daily, seeing as they are an increasing majority of transportation resources in various large cities. During the journeys, passengers and drivers are exposed to a specific complex EMF, with a dominant ELF component emitted by the driving systems and their supply installations, and an RF component emitted by various wireless communications systems (e.g., Wi-Fi routers located often inside vehicles, handsets of mobile communications used by passengers, and mobile communication BTS located outside vehicles). Depending on the location of the electric equipment inside the EVs, a higher exposure to EMF may affect passengers, or in some cases drivers.

Investigations into SMF, ELF and RF EMF emitted by various electrical equipment associated with the use of EV urban transportation showed that their levels, considered separately, comply with the limits provided by international labor law and guidelines aimed at protecting against the direct effects of short-term influence on humans of EMF of a particular frequency range (set up to prevent thermal load or electrical stimulation in exposed tissue) [12,13,17,20,21,22]. International guidelines and labor law do not provide rules on how to evaluate simultaneous exposure at various frequency ranges (e.g., SMF together with ELF and RF). This needs also specific attention, given that electronic devices and systems used inside EVs need to have sufficient electromagnetic immunity to ensure that their performance is not negatively affected by the impact from EMF emitted by the use of EVs.

Considering the chronic nature of exposure to EMF in EVs (in particular with respect to potential exposure to drivers when various EMF sources are located near their cabins), and the potential specific risks from exposure to EMF of complex composition in time and frequency domains, there is a need to collect research data on the complex characteristics of EMF exposure related to the use of EVs in public transportation and the associated health outcome in chronically exposed workers, as well as decreasing the level of their exposure by applying relevant preventive measures (e.g., locating indoor Wi-Fi routers, and other such electrical equipment, away from the driver’s cabin) [17,23,42,43,44].

## Figures and Tables

**Table 1:** Parameters of magnetic field recorded in urban electric vehicles.

Measurement Location	Magnetic Flux Density (B_RMS_, µT)									
	Trams		Trolleys		PEV Buses		PEV Passenger Car ^(1)^		HEV Passenger Car ^(2)^	
	Close to DC/AC Equipment	Distant Locations	Close to DC/AC Equipment	Distant Locations	Close to DC/AC Equipment	Distant Locations	Front Seats	Rear Seats	Front Seats	Rear Seats
	(N = 10)	(N = 20)	(N = 10)	(N = 10)	(N = 37)	(N = 65)	(N = 8)	(N = 8)	(N = 8)	(N = 8)
Median value	0.48–0.85	0.05–0.20	0.03–1.00	0.03–0.29	0.03–2.6	0.08–1.7	0.04–0.10	0.17–0.30	0.05–0.12	0.12–0.73
Maximum value	1.8–20	0.18–2.9	4.9–33	0.57–1.7	4.8–28	0.55–2.2	0.95–1.3	1.3–1.5	0.76–1.2	1.9–16

Notes: measurements of the root-mean-square (RMS) value of magnetic flux density, B_RMS_, in the measurement frequency range 40–800 Hz, with a 1.5 s sampling rate; N—number of EV journeys assisted by magnetic field recordings; DC/AC—direct current/alternating current; PEV—plug-in electric vehicle; ^(1)^ battery packs were located under the vehicle cabin, and the DC/AC power inverter and AC driving engine—battery packs in its rear part; ^(2)^ batteries and DC/AC power inverter were located in the rear part of the vehicle, and AC driving engine in the front part.
